# Respiratory syncytial virus elicits enriched CD8^+^ T lymphocyte responses in lung compared with blood in African green monkeys

**DOI:** 10.1371/journal.pone.0187642

**Published:** 2017-11-09

**Authors:** Hualin Li, Cheryl Callahan, Michael Citron, Zhiyun Wen, Sinoeun Touch, Morgan A. Monslow, Kara S. Cox, Daniel J. DiStefano, Kalpit A. Vora, Andrew Bett, Amy Espeseth

**Affiliations:** Department of Infectious Diseases and Vaccines, MRL, Merck & Co., Inc., West Point, PA, United States of America; University of Iowa, UNITED STATES

## Abstract

Respiratory syncytial virus (RSV) is a leading cause of serious lower respiratory tract disease in young children and older adults throughout the world. Prevention of severe RSV disease through active immunization is optimal but no RSV vaccine has been licensed so far. Immune mechanisms of protection against RSV infection in humans have not been fully established, thus a comprehensive characterization of virus-specific immune responses in a relevant animal model will be beneficial in defining correlates of protection. In this study, we infected juvenile naive AGMs with RSV A2 strain and longitudinally assessed virus-specific humoral and cellular immune responses in both peripheral blood and the respiratory tract. RSV viral loads at nasopharyngeal surfaces and in the lung peaked at around day 5 following infection, and then largely resolved by day 10. Low levels of neutralizing antibody titers were detected in serum, with similar kinetics as RSV fusion (F) protein-binding IgG antibodies. RSV infection induced CD8^+^, but very little CD4^+^, T lymphocyte responses in peripheral blood. Virus-specific CD8^+^ T cell frequencies were ~10 fold higher in bronchoaveolar lavage (BAL) compared to peripheral blood and exhibited effector memory (CD95^+^CD28^-^) / tissue resident memory (CD69^+^CD103^+^) T (T_RM_) cell phenotypes. The kinetics of virus-specific CD8^+^ T cells emerging in peripheral blood and BAL correlated with declining viral titers, suggesting that virus-specific cellular responses contribute to the clearance of RSV infection. RSV-experienced AGMs were protected from subsequent exposure to RSV infection. Additional studies are underway to understand protective correlates in these seropositive monkeys.

## Introduction

Human respiratory syncytial virus (RSV) has been identified as the leading cause of severe respiratory disease in infants [1]. Severe RSV illness commonly occurs among infants with primary infection in the first year of life, and most infants have experienced a primary RSV infection by age two [2]. Globally, it is estimated that RSV infection results in 64 million acute respiratory infection cases and 160,000 deaths annually [1]. While healthy young adults generally only suffer common cold symptoms and are at low risk of severe disease, adults with underlying diseases, such as COPD or asthma, or those who are immune-compromised, are also at a high risk of developing severe RSV infection [3–5]. In addition, RSV has been recognized in recent years as a significant problem in debilitated and elderly persons and infection may lead to cardiac failure and secondary bacterial pneumonia [5, 6]. RSV can cause severe lower respiratory complications in older adults, resulting in respiratory failure, prolonged hospitalization, and high mortality similar to seasonal influenza [6].

Despite the increased appreciation of the large global impact of RSV disease, there remains no licensed active vaccine. Passive immunotherapy with RespiGam (RSV immune globulin) [7] and the RSV fusion (F) protein-specific humanized monoclonal antibody palivizumab (Synagis) [8] were approved to be used in infants at high risk of developing severe RSV lower respiratory tract infection (LRTI). However, a safe and effective vaccine would be a more cost effective solution for the prevention of RSV in at risk populations. Efforts to develop a safe and effective RSV vaccine have been largely daunted by the failure of a formalin-inactivated RSV (FI-RSV) vaccine in a clinical trial ~50 years ago [9]. Despite a recent increase in interest, investment, and progress towards development of RSV vaccines for infants and / or the elderly [10, 11], a number of challenges remain for the development of an effective RSV vaccine, including major unanswered questions surrounding the human immune responses and protection correlates to an RSV infection. Epidemiological and human challenge studies have pointed to a variety of factors associated with protection from RSV, including neutralizing antibodies in either serum or nasal secretions, or nasal mucosal IgA / IgG specific to RSV or RSV fusion (F) protein [12–16]. Furthermore, RSV neutralizing antibody titers and F-binding antibody titers were also reported to be inversely correlated with RSV-associated hospitalization [17]. On the other hand, elderly adults were found to have similar levels of RSV neutralizing antibody titers but lower frequencies of RSV-specific cellular responses compared to young adults, suggesting that deficient T cell responses may contribute to severe RSV diseases in elderly [18]. While these studies have provided insight into the factors associated with protection from RSV infection in older children and adults, little is known about the immune correlates of protection in infants beyond a role of antibody as suggested by the success of immune prophylaxis. The Afrcian green monkey model offers the opportunity to perform a comprehensive characterization of the immune response to RSV infection in a naïve primate.

Naïve African green monkeys (AGM) are semi-permissive to RSV infection [19] and are an important animal model for RSV studies on viral pathogenesis, vaccine development and antiviral research. The AGM model of RSV infection offers two key benefits over human experimental models. First, human RSV experimental infection studies can only be conducted in adults and all adults are RSV-experienced, limiting the value of the human challenge model to characterize immune correlates for naïve infants. In contrast, juvenile AGMs are RSV seronegative and provide an ideal opportunity for study of the primary host immune responses to RSV infection. Second, RSV infects through the upper respiratory tract mucosa and the infection is typically confined to the respiratory compartment. Therefore, it is critical to study host responses to RSV at respiratory mucosal sites in addition to those in peripheral blood for a full characterization of protective immunity. While mucosal samples may be difficult to obtain from humans, they are easier to collect from nonhuman primates.

In this study we infected RSV seronegative juvenile AGMs with the RSV A2 strain and conducted a comprehensive assessment of virus-specific immune responses, including humoral and cellar immune responses, in peripheral blood, in the nasal mucosa, and in lung. AGMs showed similar kinetics of viral shedding compared to human, suggesting that AGMs are a valid model for studying primary RSV infection. RSV-infection induced low levels of humoral responses in peripheral blood and at mucosal sites, as well as very low levels of CD4^+^ T cell responses. In contrast, RSV-infection induced clearly detectable levels of CD8^+^ T cell responses in peripheral blood and importantly, RSV-specific CD8^+^ T cell responses were ~10 fold enriched in lung. The kinetics of the emergence of RSV-specific CD8^+^ T cell responses in blood and lung correlated with a decline in viral loads, suggesting that T cell responses contributed to the clearance of RSV infection. These RSV seropositive AGMs became resistant to RSV re-challenge. Further investigations are underway to determine the immune mechanism(s) of protection against RSV infection in seropositive AGMs.

## Material and methods

### Animals and virus

African green monkeys (*Chlorocebus sabaeu*, AGM) were domestically bred, raised and maintained at New Iberia Research Center (NIRC) of University of Louisiana at Lafayette, New Iberia, LA, USA. RSV seronegative (RSV neutralizing titers <4) juvenile monkeys (at 12–24 months of age) were used in this study. The animal studies were approved by the University of Louisiana at Lafayette Institutional Animal Care and Use Committee (IACUC) and conducted in accordance with animal care guidelines.

RSV strain A2 (ATCC VR-1540) and Long (ATCC VR-26) stocks were grown in Hep2 cells. Hep2 cells were cultured in Eagle minimum essential medium (EMEM) containing 10% fetal bovine serum (FBS), 2 mM L-glutamine, 50 μg/ml Gentamicin, 25 μg/ml Amphotericin B and 1% Penicillin-Streptomycin. Cell free virus was harvested at 5 to 7 days post infection, flash frozen on liquid nitrogen and stored at -70°C.

### RSV infection of AGMs and sample collection

All animals were socially housed prior to the start of the study. Animals were paired housed before RSV challenge and singly housed upon RSV challenge. The dimensions of the cage for singly housing are 4.3 (floor area/animal, foot square) and 30 (height, inch). For paired housing, two of these cages were placed side by side so both animals have access to both cages. Each animal’s immediate holding cage was cleaned daily. Animals were provided with object(s) to manipulate or explore. Harlan Teklad Monkey Chow, or its equivalent, was provided daily in amounts appropriate for the size of the animal. The basic diet was supplemented with fruit and novel treats including small quantities of fresh fruits, nuts, or seeds, 2 to 3 times weekly as part of the site's environmental enrichment program. Tap water was provided ad libitum via automatic watering device. No contaminants are known to be present in the food or water which would interfere with the results of this study. Food was withheld at least 2–3 hours on days of study procedures to insure safe sedation and was offered upon recovery from sedation.

In a pilot study, RSV seronegative animals (N = 8) were anesthetized with Ketamine (10 mg/kg), and challenged with 2 × 10^5.5^ plaque forming unit (pfu) of RSV A2 strain. The challenge virus was administered by intranasal and intratracheal inoculation, 1 ml by each route. To determine RSV replication / shedding in AGMs, nasopharyngeal (NP) swabs and bronchoalveolar lavage (BAL) samples were collected at days 0, 3, 5, 7, and 10 following infection. Animals were sedated with Telazol (4–6 mg/kg) and supplementation of Ketamine (5 mg/kg) if necessary when NP and BAL samples were taken. The NP swab samples were collected by gently rubbing two areas of the oropharynx region using a Darcon swab and placing the tips in a solution containing Hank’s balanced salt solution (HBSS) with 10% in-house made SPG buffer (2.18M sucrose, 0.038M KH2PO4 (mono-basic), 0.072M K2HPO4 (di-basic), and 0.049M Na Glutamate; pH 7) and 0.1% gelatin. To collect BAL samples, approximately 5 ml HBSS was infused directly into the lung and aspirated via a sterile French catheter and syringe. Recovered samples were supplemented with 0.1 volume of 10 × SPG and 0.1 volume of 1% gelatin, aliquoted, flash frozen and stored at -70°C. NP swabs and BAL were also collected at day 14 and 28 to determine RSV infection-induced humoral / cellular immune responses. Cell pellets from freshly collected BAL were used to determine T lymphocyte responses and supernatant was used to determine antibody responses. Peripheral blood was collected to determine peripheral RSV-specific humoral and cellular responses.

In a second study, another group of RSV seronegative animals (N = 8) were infected with RSV and peripheral blood and BAL samples were collected at days 0, 7, 9, 14, 21, and 28 following infection to determine the magnitude and phenotype of RSV-specific T lymphocyte responses.

Animals were observed twice daily throughout the study for any abnormal clinical signs, signs of illness or distress. All animals were returned to the colony at NIRC at the end of the study with negative RSV shedding at NP confirmed.

### Human sera

Serum samples from healthy adult human (18–60 years old) were purchased from Biological Specialty Corporation in Colmar, PA, USA.

### RSV plaque forming assay

The RSV plaque forming assay was performed as described (Z. Wen, D. Casimiro, and D. DiStefano. Unpublished conference presentation). Briefly, HEp2 cells at concentration of 1.2×10^6^ cells / ml in EMEM medium (EMEM supplemented with 2% FBS and 2mM glutamine) were seeded at 50 μl per well into round-bottom 96 well plates. Seventy-five microliters of viral inoculum or 2-fold serially diluted samples were added to each well. Samples were mixed well with cells and then incubated for 1 hour at 37°C before the plate was centrifuged at 300×g for 10 minutes for better host cell settlement. One hundred fifty microliters of EMEM medium supplemented with 1% methycellulose (Sigma-Aldrich) was overlaid in each well to prevent viral spread to neighboring cells. Plates were incubated at 37°C with 5% CO2 for 3 days. Cells were then washed with phosphate buffered saline (PBS) and fixed with ice-cold 80% acetone (Sigma-Aldrich) for 10–20 minutes. The plate was then allowed to dry for 20 min before washing with PBS supplemented with 0.05% tween (PBST). Fixed cells were stained with an in-house mouse anti-RSV F antibody (1.25 μg/ml) and an anti-nucleoprotein monoclonal antibody (1.25 μg/ml). These two antibodies were incubated with the fixed cells for 1 hour before anti-mouse IgG Alex488 conjugated secondary antibody (Invitrogen) was added (1:500 diluted). Unbound secondary antibody was washed off after 1 hour of incubation. Plates were analyzed for image capturing and automated counting by EnSight imager reader 2.02 (PerkinElmer).

### RSV quantitative reverse transcriptase PCR (RT-qPCR) assay

RSV RT-PCR was performed as follows. Each NP swab was eluted with 300 μl of PBS and RSV RNA was extracted using a Maxwell® 16 Viral Total Nucleic Acid Purification Kit (Promega) according to manufacturer’s instruction. Briefly, 300 μl of NP eluate was mixed with 330 μl of lysis solution and heated for 10 minutes. The lysates were then added to the cartridge and loaded into the Maxwell® 16 instrument for RNA purification. Purified RNA was tested in the RSV RT-qPCR assay using a Quantitect® Probe RT-PCR kit (Qiagen) with RSV Nucleoprotein (N) gene being the target. Primers were designed to the conserved region of the N gene and the probe for RSV A contained the fluorescent reporter dye 6-carboxyfluorescein (FAM) at the 5′-end and the fluorescent quencher dye 6-carboxytetramethylrhodamin (TAMRA) at the 3′-end. Each sample was tested in duplicate. The sensitivity of the assay is 10 copies of RSV RNA.

### RSV F-specific IgG ELISA assay

Immulon® 2HB microtiter plates (NUNC) were coated with 2 μg/ml recombinant RSV F proteins (Pre F and Post F respectively) made in-house, and incubated at 4°C overnight. The plates were then washed and blocked for 1 hour with PBST containing 3% non-fat milk (blocking buffer) at room temperature. Test samples were serially diluted 4-fold in blocking buffer (starting at 1:50 dilution for serum samples and 1:4 for mucosal samples), transferred to the RSV F coated plates, and incubated for 2 hours at room temperature. Following three washes with PBST, HRP conjugated anti-human IgG secondary antibody (Invitrogen) diluted 1:2,000 in blocking buffer was added to the plates and incubated for an additional 1 hour. Plates were washed again and developed with SuperBlu Turbo TMB (Virolabs) in the dark. The reaction was stopped after 5 minutes and absorbance was read at 450 nm on a VersaMax ELISA microplate reader (Molecular Devices). Titers are reported as the reciprocal of the last dilution that is 3 fold greater than the background.

### RSV F-specific IgA ELISA assay

The IgA titers in serum and mucosal samples were quantified using a direct binding ELISA on the Meso Scale platform (Meso Scale Discovery, MSD). Briefly, 96-well standard Meso Scale plates were coated with 0.2 μg/ml recombinant RSV F protein made in-house or ovalbumin (Worthington Biochemical) protein at 4°C overnight. The plates were then washed and blocked for 1 hour with blocking buffer at room temperature. Sera or mucosal samples were serially diluted 2-fold in HISPEC buffer (Bio-Rad), transferred to the RSV F protein coated plates and incubated for 1 hour at room temperature. After washing, SULFO-TAG (MSD) conjugated secondary antibody (Jackson ImmunoResearch) diluted to 1:1,000 in HISPEC buffer was added to the plates and incubated for 1 hour at room temperature. Plates were washed again and 1× Read Buffer T (MSD) was added to the plates. Plates were immediately read on a Sector S 600 plate reader (MSD). RSV F-specific ECL values were background adjusted by subtracting ovalbumin values. Titers are expressed as the reciprocal of the last dilution that was 3 fold greater than the background.

### RSV microneutralization (MN) assay

RSV MN assay was performed as described with modifications [20]. All sera were treated at 56°C for 30 min to inactivate complement prior to testing in the neutralization assay [21]. Two-fold serial dilutions of AGM or human serum samples were prepared in EMEM containing 2% FBS starting at 1:4 dilution. Diluted serum was added in duplicate to 96-well plates and mixed with RSV Long strain (100 pfu/ml) in 100 μl total volume. The mixture of virus and serum samples was incubated for 1 h at 37°C with 5% CO_2_. Following incubation, Hep-2 cells at a concentration of 1.5 × 10^4^ cells per well were added. The plates were incubated for 3 days at 37°C with 5% CO_2_. The cells were then washed and fixed with 80% acetone for 15 minutes. RSV infected cells were then immunostained. Briefly, in house-made RSV F- and N-specific monoclonal antibodies were added to the test plates with fixed cells and incubated for 1 hour at room temperature. After washing, biotinylated goat anti-mouse IgG was added and incubated for 1 hour. The plates were washed again and developed by a dual channel near infrared detection (NID) system. Infrared dye-Streptavidin to detect RSV specific signal and two cell stains for assay normalization were added to the 96-well plates and incubated for 1 hour in the dark. Plates were washed, dried in the dark for 20 minutes, and read on the Licor Aerius® Automated Imaging System utilizing a 700 channel laser for cell normalization and an 800 channel laser for detection of RSV specific signal. The 800/700 ratios were calculated and serum neutralizing titers (IC50) were determined by four parameter curve fit in GraphPad Prism 7 software. The neutralization titers determined by the microneutralization (MNT) assay and the plaque reduction assay (PRNT) assay [17] were not statistically different.

### Multi-parameter intracellular staining (ICS) assay

The ICS assay was performed essentially as described [22]. Peripheral blood mononuclear cells (PBMC) were isolated from whole blood by Ficoll gradient sedimentation. BAL fluid lymphocytes were collected by centrifuging lavage fluid at 250×g for 10 minutes. PBMC (1.0×10^6^) or BAL fluid lymphocytes were incubated for 6 hours at 37°C with medium, 20 ng/ml phorbol myristate acetate and 1.25 μg/ml ionomycin (Sigma-Aldrich), or 2 μg/ml RSV F (in 2 subpools), G, N, M, M2 peptide pools (JPT Peptides Technologies). Cultures contained Brefeldin A at 2.5 μg/ml (Sigma-Aldrich) and 1 μg/ml mAb against human CD49d (clone 9F10; BD Biosciences). The cells were then stained with predetermined dilutions of mAbs against CD3 (SP34-2; PE), CD4 (L200; PECF594), CD8 (SK1; APC-H7), and the LIVE/DEAD® fixable dead cell stains (Invitrogen) at room temperature for 30 minutes. Cells were then permeabilized using fixation / permeabilization solution (BD Biosciences) and stained with antibodies specific to cytokines including gamma interferon (IFN-γ; B27; FITC), interleukin-2 (IL-2; MQ1-17H12; APC), and tumor necrosis factor alpha (TNF-α; Mab11; PE-cyanine 7 [PE-Cy7]). For phenotyping of RSV-specific T cells, mAbs against CD3 (SP34-2; BUV395), CD4 (L200; BV786), CD28 (L293; PE), CD95 (DX2; PE Dazzle 594; Biologend), CD69 (FN50; PE-Cy5; Biolegend), Ki67 (B56; BV510), CD103 (Ber-ACT8, APC; Biologend), and IL-2(MQ1-17H12; APC-R700) were used to facilitate the evaluation of T lymphocyte subsets and phenotypes. MAbs were purchased from BD Biosciences unless specified. Stained samples were fixed with 1.0% paraformaldehyde solution and evaluated with a LSRII flow cytometer (BD Biosciences). Approximately 300,000 to 500,000 events were collected per sample. Flow data was analyzed using FlowJo software (Tree Star, Inc.). Background responses were typically below 0.01% of the gated CD4^+^ or CD8^+^ peripheral T lymphocytes and below 0.2% of the gated CD8^+^ BAL fluid lymphocytes. Cytokine values were analyzed following subtraction of the background (mock stimulation). Polyfunctionality of CD8^+^ T lymphocytes was determined using SPICE software (NIAID, NIH, http://exon.niaid.nih.gov/spice) after Boolean gating cytokine positive cells in FlowJo software.

### Statistical analyses

Statistical analysis was carried out using GraphPad Prism 7 software. A p value <0.05 indicates statistical significance.

## Results

### AGMs exhibit similar kinetics as humans for viral replication and viral shedding following RSV infection

**Fig 1:**
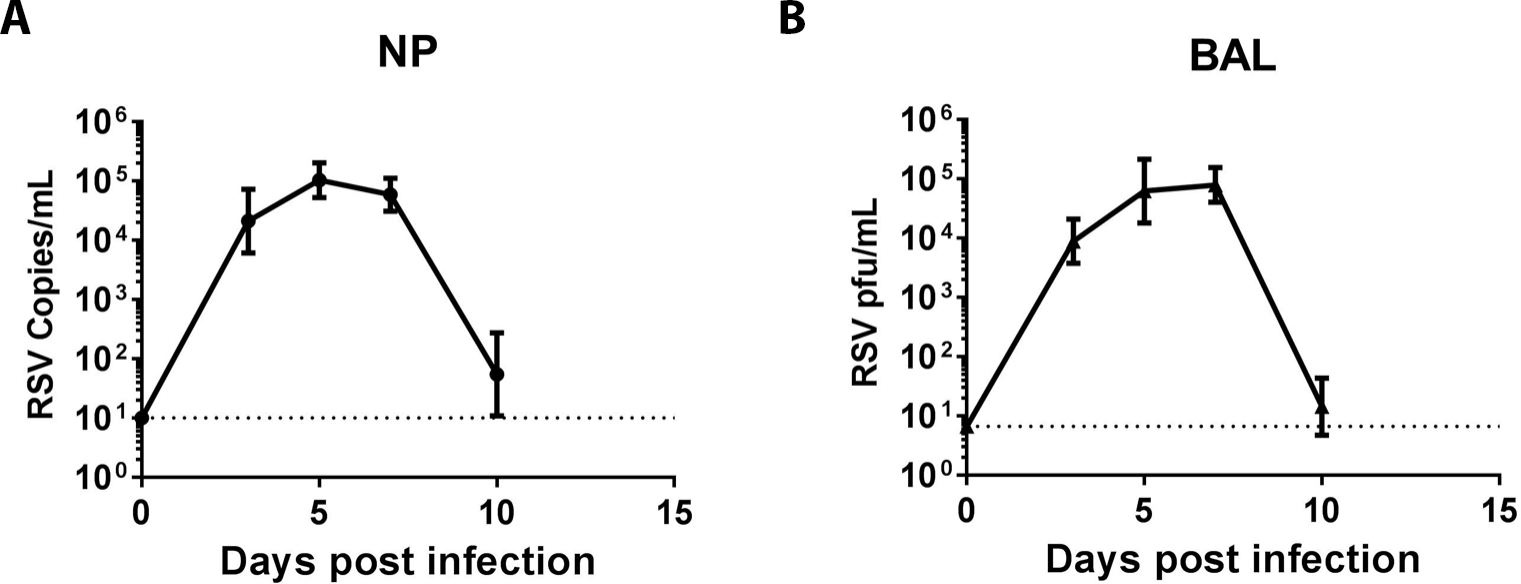
RSV viral replication in AGMs following infection. Eight AGMs were infected with RSV A2 strain through intranasal and intratracheal inoculation. RSV viral shedding at nasopharyngeal (NP) mucosal surfaces and viral replication in lung (BAL) were determined at 0, 3, 5, 7, and 10 days following infection. (A). RSV viral loads in NP swab elutes were determined by RSV RT-qPCR (Geomean with 95% confidence interval (CI)). (B). RSV viral loads in BAL were determined by RSV plaque forming assay (Geomean with 95% CI). Dashed lines represent limit of detection.

A pilot study to assess RSV infection in AGM was carried out in a cohort of eight seronegative juvenile AGMs, which were inoculated with 2 ml of 10^5.5^ pfu/ml RSV A2 strain delivered both intranasally (1 ml) and intratracheally (1 ml). Viral shedding at NP mucosal surfaces was assessed from eluates of NP swabs by RT-qPCR. RSV infection in lung was assessed by plaque assay from BAL fluid. All eight monkeys were successfully infected as evidenced by the presence of viral mRNA in NP eluates and replication competent virus in lung samples (Fig 1). Viral shedding in the NP peaked at around day 5 following infection (Fig 1A). Viral replication in lung exhibited similar kinetics with peak viral loads from days 3–7 and then quickly declined thereafter (Fig 1B). Both NP viral shedding and lung viral replication were largely resolved by day 10. The kinetics and duration of RSV shedding / replication in AGMs were similar to that observed in the previous AGM studies [19, 23] and in humans experimentally infected with RSV [24, 25]. No symptoms were observed in these animals post RSV infection.

### Primary RSV infection induced low levels of humoral immune responses in peripheral blood and at mucosal sites

**Fig 2:**
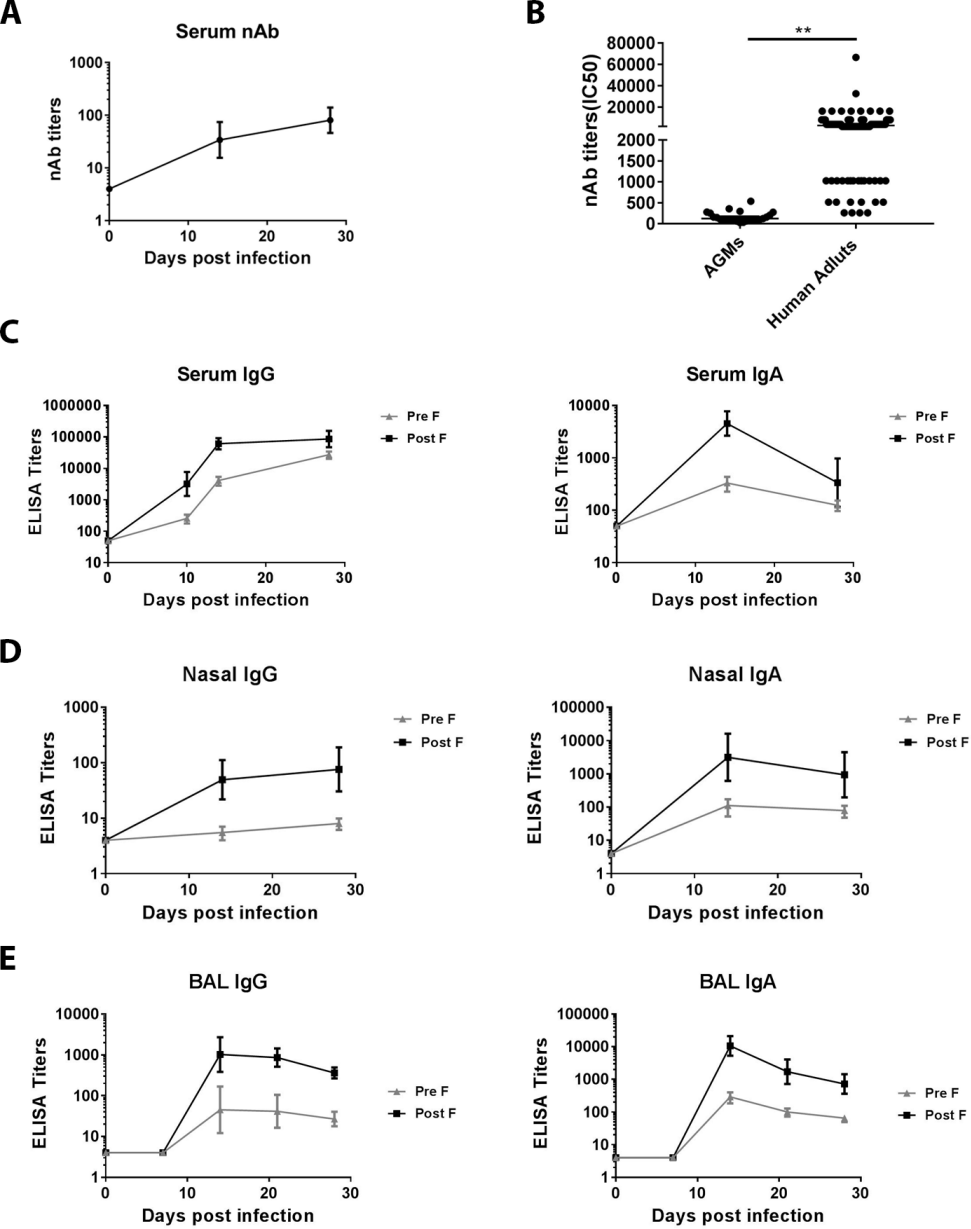
RSV humoral immune responses in AGMs following infection. Antibody responses in AGMs were determined at 0, 7, 14, 21, and 28 days following RSV infection. (A). Serum neutralizing titers (Geomean with 95% CI) to RSV Long strain were determined by RSV MN assay. (B). Serum neutralizing antibody titers in AGMs (N = 24) at day 28 post infection were compared to those a collection of human adults (18–60 years old; N = 98). **, p<0.01, student test. (C). Serum IgG and IgA antibody responses to RSV A2 F protein (Pre F and Post F proteins respectively) were determined by ELISA. Mucosal antibody responses specific to RSV F proteins were also determined for nasal mucosa using NP swab eluates (D) and lung using BAL samples (E) by ELISA. All ELISA titers are expressed as Geomean with 95% CI.

We assessed the kinetics of RSV-specific humoral immune responses in AGMs in peripheral blood as well as in NP and in lung post infection. Serum samples were tested for neutralizing activity against the RSV Long strain. All monkeys had detectable levels of serum neutralizing antibodies by 14 days following infection and neutralizing antibody titers increased slightly by day 28 (Fig 2A). Neutralizing antibody titers in AGMs at day 28 post infection (range: 25–536; median: 103) were comparable to those reported in infants following primary infection [26]. In contrast, RSV neutralizing antibody titers in healthy adult human (N = 98; range: 256–66,536; median: 4096) were significantly higher, probably due to repetitive exposure to RSV virus during their lifetime (Fig 2B).

RSV fusion protein (F) is a major target of neutralizing activity in human sera and is also the target of palivizumab as well as the target of various RSV vaccine candidates in development [27, 28]. Moreover, on the surface of the virus, F protein exists in a metastable pre-fusion (Pre F) conformation that, during the infection process, rearranges to a more stable post-fusion (Post F) form, in the process enabling viral entry into the host cell [29]. We therefore assessed IgG and IgA antibody titers to F protein in both Pre F and Post F conformations in serum and at mucosal sites.

RSV F-specific IgG titers were ~ 1 log higher than IgA in serum, for both Pre F- and Post F-binding antibodies (Fig 2C). Serum RSV F-specific IgG antibody responses mirrored similar kinetics to neutralizing antibody titers rising through day 28 (Fig 2A and 2C left panel). In contrast, serum IgA exhibited different kinetics than IgG / neutralizing titers, with a peak response at day 14 and a sharp decline by day 28 (Fig 2C right panel). For both serum IgG and IgA, Post F-binding titers were around 10-fold higher than those of Pre F-binding. While Pre F-binding IgG titers are normally comparable or slightly higher than Post F-IgG titers in human serum [30] and Pre F-specific antibodies are the major contributors to RSV neutralization activity [30–32], the reversed ratio of Pre F vs Post F titers in AGMs could reflect the difference of lab-prepared viral inoculum used in this study compared to transmissible virus in human.

We also determined RSV F-specific antibody titers in samples taken from respiratory mucosa. Unlike in serum, RSV F-specific IgA titers are ~1 log higher than IgG in nasal secretions (Fig 2D), highlighting the difference between systemic and mucosal antibody immune responses to a mucosal infection. Similar to serum, both IgA and IgG titers are higher to Post F than to Pre F. Additionally, we tested RSV F-specific antibody titers in BAL fluid. Analogous to nasal secretions, BAL fluid exhibited ~1 log higher titers of IgA than IgG, for both Pre F and Post F, with Post-F titers 10x higher than Pre-F (Fig 2E). Nevertheless, mucosal RSV F-specific IgA titers exhibited similar kinetics to mucosal IgG, without the abrupt drop from day 14 to day 28 observed with serum IgA.

### RSV infection induced virus-specific CD8^+^ T lymphocyte responses in peripheral blood and lung

**Fig 3:**
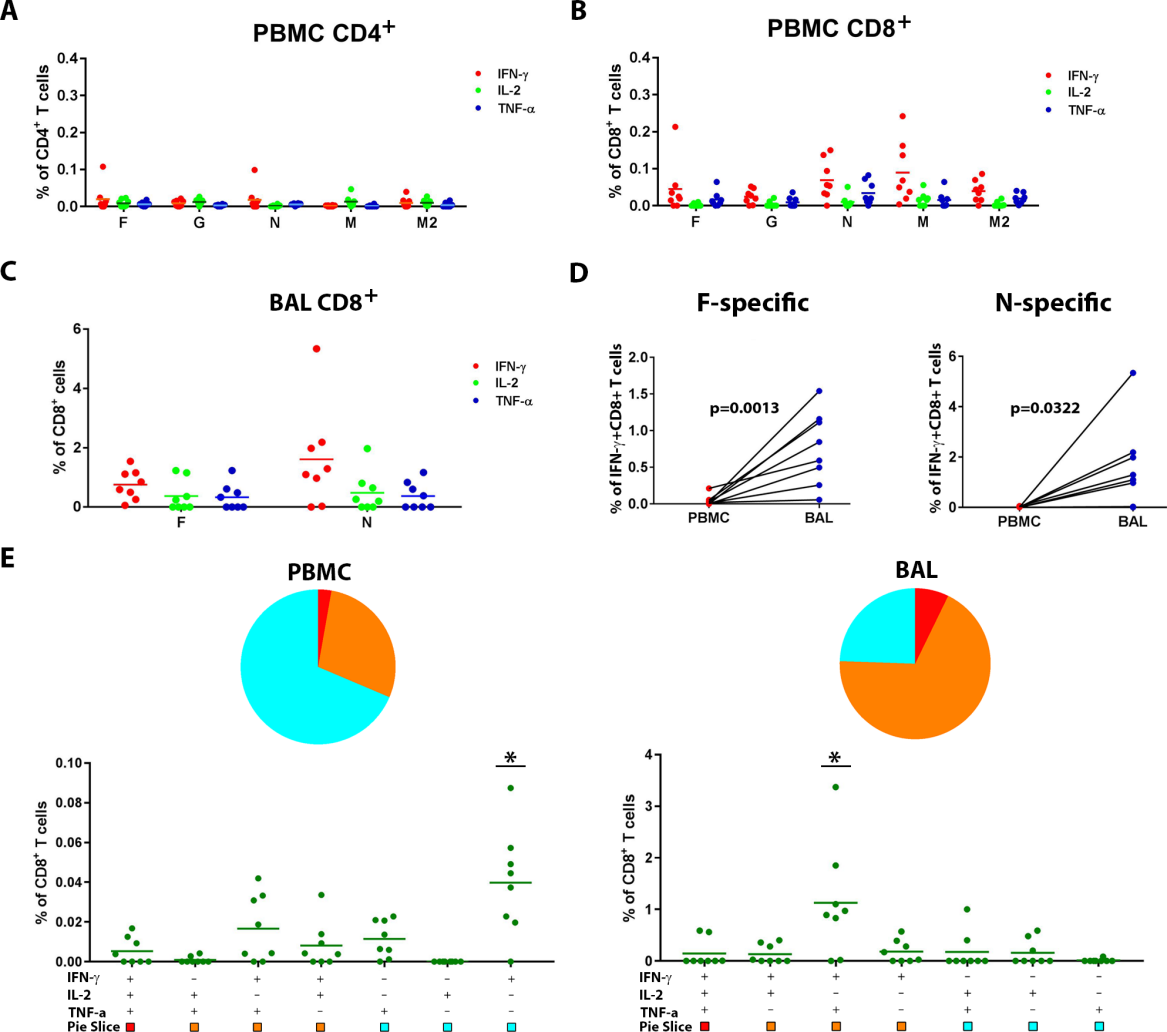
RSV cellular immune responses in AGMs following infection. RSV-specific cellular immune responses in AGMs at 28 days following infection were determined by multiparameter flow cytometry. Cytokine secretions including IFN-γ, IL-2, and TNF-α of CD4^+^ (A) and CD8^+^ (B) T cells in PBMC specific to various RSV antigen stimulations are depicted (line represents mean). (C). Cytokine secretions of CD8^+^ T cells in lung in response to RSV antigens F and N (line represents mean) were depicted. (D). Comparison of RSV F- and N-specific CD8^+^ T cell responses (represented by IFN-γ-secreting CD8^+^ T cells) in PBMC and in lung (N = 8) with p value for paired t test was depicted. (E). Polyfunctionality of RSV-specific CD8^+^ T cell responses in PBMC and BAL (N = 8) were analyzed by Boolean gating using FlowJo software and graphed using SPICE software. The percentage of RSV N-specific CD8^+^ T cells in PBMC and lung secreting one cytokine, 2 cytokines, or 3 cytokines at the same time are depicted respectively in the pie charts. The absolute magnitude of each population secreting individual cytokines, or combinations of two or three cytokines, are depicted in the scattered dot plots below the pie charts (line represent mean). *, p<0.05, one-way ANNOVA compared to the other populations.

To assess RSV infection-induced cellular immune responses in peripheral blood, PBMC were stimulated with various peptide pools (15-mer peptides, overlapping by 11 amino acids) of RSV antigens including fusion protein F, attachment protein G, matrix protein M & M2, and nucleocapsid protein N. Peptide-stimulated cells were incubated for 6 hours in the presence of brefeldin A and then analyzed for cytokine secretions by multi-parameter flow cytometry. Gating strategy and representative plots for CD4^+^ and CD8^+^ T cell cytokine secretions were shown in S1 Fig. RSV-specific CD4^+^ T lymphocyte responses were very low or undetectable for all of the antigens tested, with positive responses only observed in one animal (Fig 3A). In contrast, all animals mounted positive CD8^+^ T lymphocyte responses to RSV proteins (Fig 3B). The magnitude of CD8^+^ T cell responses specific to internal proteins (N, M, M2) was greater than those specific to surface proteins (F, G), which is in agreement with results from human challenge studies [33]. To study the mucosal immune response at the site of infection and viral replication, we collected BAL fluid and isolated cells for T-cell response evaluation with *in vitro* RSV peptide stimulation. Due to the limited number of cells isolated from BAL, we only tested one surface protein, F, and one internal nucleocapsid protein, N. The majority of lymphocytes isolated from BAL were CD8^+^ T lymphocytes, making analysis of CD4^+^ T cells challenging. We therefore focused on analyzing CD8^+^ T lymphocytes from BAL. Most of the animals showed positive CD8^+^ T cells responses in BAL upon stimulation with RSV F and /or N peptides (Fig 3C). Variations of CD8^+^ T cell responses in PBMC and / or BAL among these monkeys could have reflected the outbred nature of these animals and /or different kinetics of T cell responses in these animals. Compared to responses in PBMC, CD8^+^ T cell responses in BAL were ~10 fold enriched (Fig 3C and 3B). Indeed, magnitudes of RSV-specific CD8^+^ T lymphocyte responses in BAL were significantly higher than those in peripheral blood (p = 0.0013 and 0.0322 for F- and N-specific responses respectively, paired t-test, Fig 3D), highlighting the quantitative differences between local T cell responses and systemic T cell responses during RSV infection. As observed for PBMC, the levels of N-specific CD8^+^ T cells were higher than those specific to F protein. In addition, RSV infection-induced CD8^+^ T cell responses in PBMC appeared to be of limited polyfunctionality, with most cells secreting IFN-γ only (Fig 3E, left panel). In contrast, RSV-specific CD8^+^ T cells in BAL tend to be more polyfunctional, with a greater portion of cells secreting two or three cytokines. Most RSV-specific CD8^+^ T cells in lung secret both IFN-γ and TNF-α (Fig 3E, right panel). This indicates that mucosal T cells can be qualitatively and quantitatively different from those in peripheral blood, similar to antibody isotype responses. Furthermore, RSV-specific CD8^+^ T cells in peripheral blood and lung remained detectable at 6 months post infection (data not shown).

### Kinetics of RSV antigen-specific CD8^+^ T lymphocyte responses in AGMs following infection

**Fig 4:**
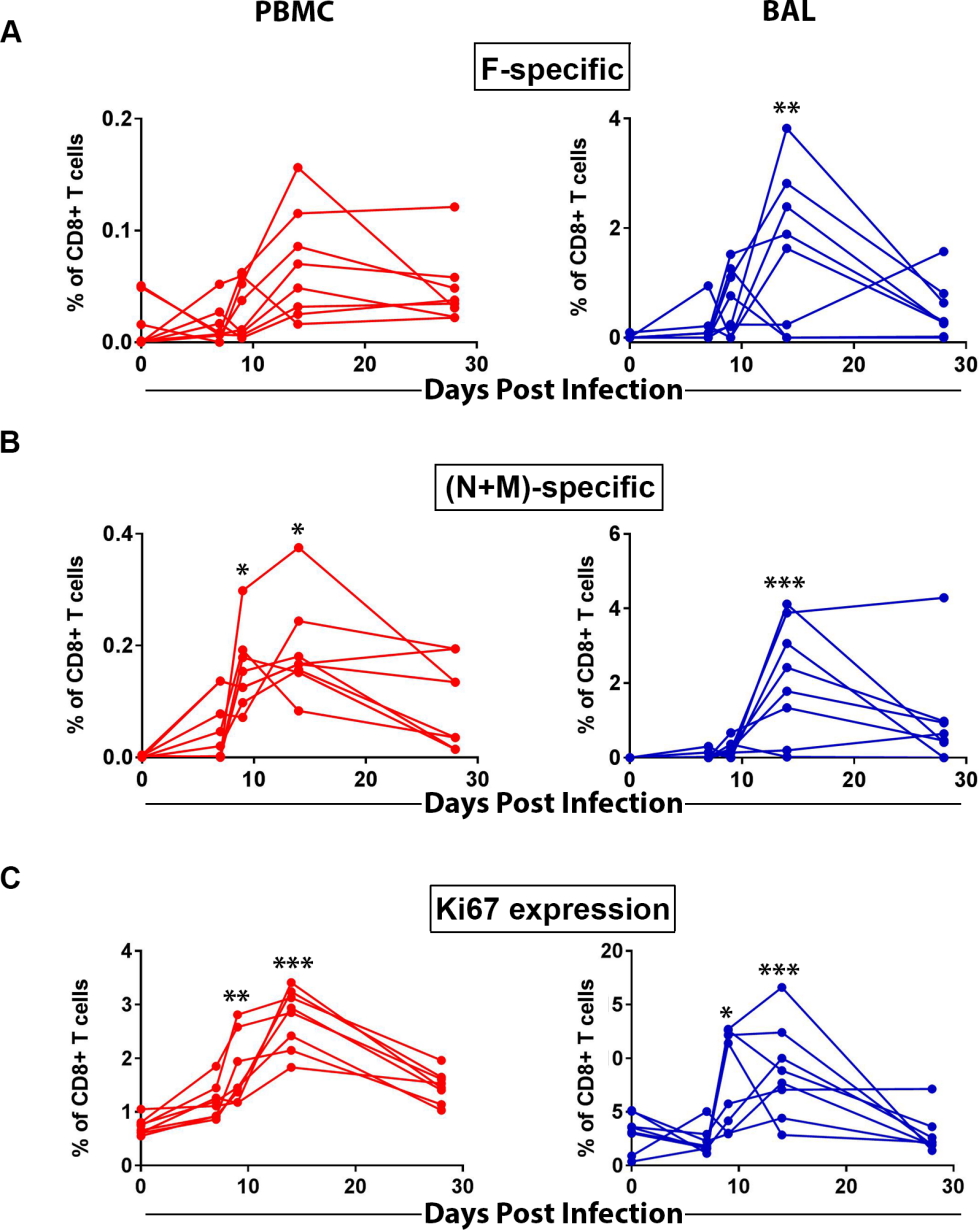
Kinetics of RSV-specific CD8^+^ T cell responses in AGMs following infection. A second group of eight AGMs were infected with RSV A2 strain and CD8^+^ T cell responses were determined by multiparameter flow cytometry with an expanded antibody panel at 0, 7, 9, 14, 21, and 28 days following infection. (A) PBMC or cells isolated from BAL were stimulated with RSV F protein overlapping peptides for evaluation of cytokine secretions. The percentage of IFN-γ-secreting CD8^+^ T cells were used to represent the magnitude of virus-specific CD8^+^ T cell responses. (B). Frequency of IFN-γ-secreting CD8^+^ T cells in responses to RSV proteins N and M (N+M) overlapping peptides stimulation in PBMC and BAL. (C) Expression of proliferation marker Ki67 in CD8^+^ T lymphocytes from PBMC or BAL. *, p<0.05; **, p<0.01; ***, p<0.001. One-way ANNOVA compared to baseline (day 0) levels.

Since the pilot study revealed more measurable virus-specific CD8^+^ T cell responses, we focused on characterizing RSV infection-induced peripheral and mucosal CD8^+^ T lymphocyte responses in AGMs, including the kinetics and phenotype of these responding cells. In our follow up study we infected another group of eight RSV naïve AGMs and assessed virus-specific CD8^+^ T lymphocyte responses from day 7 to 28 post-infection. To maximize the information we can obtain from the limited number of cells (e.g. BAL), we focused on testing both RSV F protein and a mixed pool of N and M proteins (N+M) using an expanded ICS panel. Multi-parameter ICS data were analyzed according the same gating strategy as shown in S1 Fig. RSV-specific CD8^+^ T cell responses (represented by IFN-γ-secreting CD8^+^ T cells) were detected in some monkeys as early as 7 days following infection in both PBMC and BAL (Fig 4A and 4B). All animals exhibited positive RSV-specific CD8^+^ T cell responses by day 9 (Fig 4A and 4B). The emergence of RSV-specific CD8^+^ T cells coincided with the decline of viral loads in lung and at nasopharyngeal mucosa surfaces (Fig 1), suggesting that virus-specific CD8^+^ T cells may have contributed to the clearance of local RSV infection. While some animals showed a little different kinetics, CD8^+^ T cell responses reached a peak in most animals by day 14 and then declined by day 28 (Fig 4A and 4B). We also assessed the expression levels of T cell proliferation marker Ki67 in CD8^+^ T lymphocytes. In both peripheral blood and BAL, the kinetics of CD8^+^ T cells expressing Ki67 overall mirrored those of virus-specific CD8^+^ T cells (Fig 4C).

### Phenotype of RSV-specific CD8^+^ T lymphocytes in peripheral blood and in lung

Given the quantitative and qualitative differences between RSV-specific CD8^+^ T cell responses in lung and in peripheral blood observed in the pilot study, we decided to further explore any phenotypic differences between the CD8^+^ T cells in these two compartments. Peripheral CD8^+^ T lymphocytes were divided into three subpopulations according to the expression of memory marker CD95 and costimulatory molecule CD28. CD95^-^CD28^+^ defined naïve T cells, CD95^+^ CD28^+^ defined central memory T cells (T_CM_), and CD95^+^CD28^-^ defined effector memory T cells (T_EM_), as previously reported [34]. In recent years, it has become clear that an additional memory subset, termed tissue resident memory T cells (T_RM_), resides in non-lymphoid tissue without recirculating [35–37]. The markers CD103 and CD69 are used to define T_RM_ in mouse, human and rhesus monkeys.

**Fig 5:**
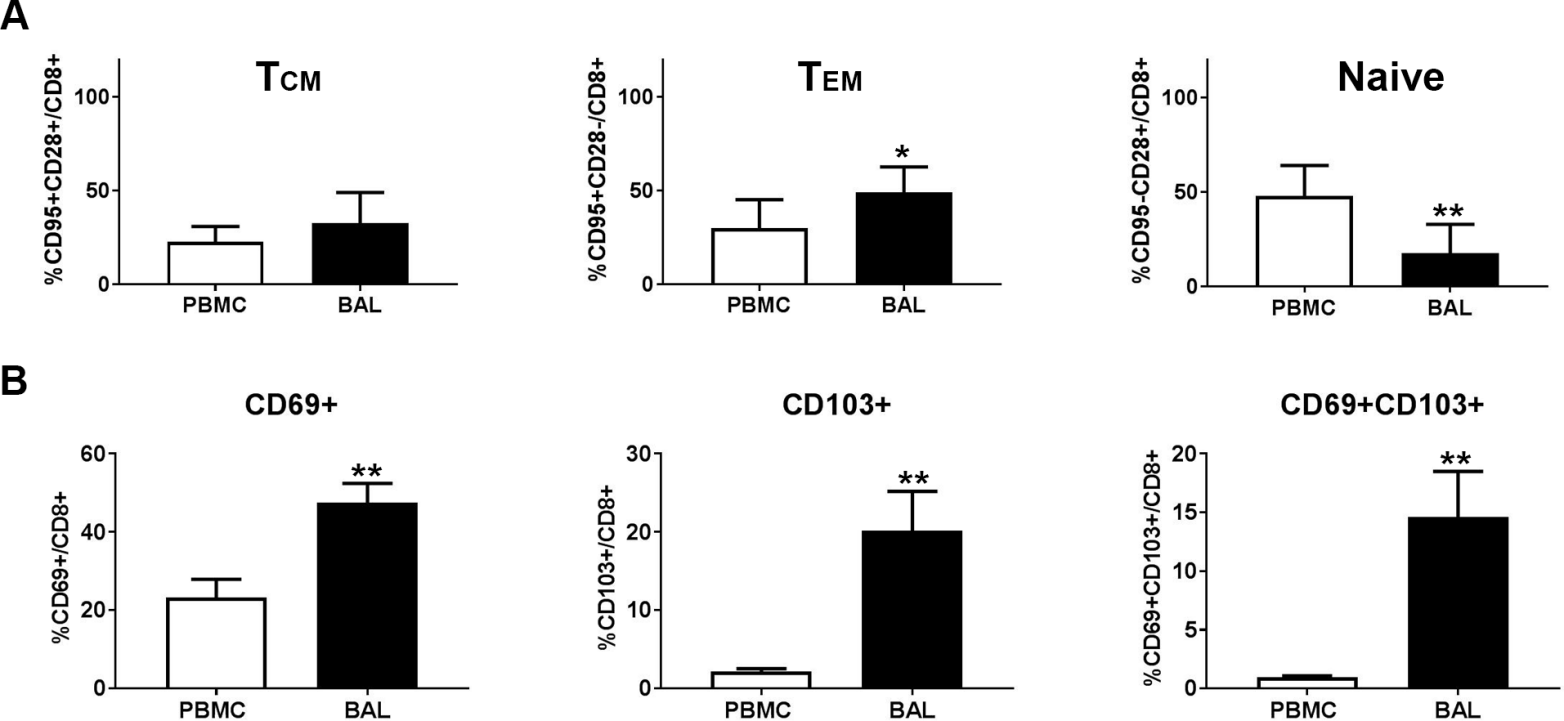
Phenotype of CD8 ^+^ T lymphocytes in PBMC and in lung. Phenotype of CD8^+^ T lymphocytes in PBMC and lung in AGMs before RSV infection were determined by multi-parameter flow cytometry. (A). The percentage of T_CM_, T_EM_ and naïve CD8^+^ T cell in PBMC and lung are depicted. CD95^+^CD28^+^ defines central memory T cells (T_CM_), CD95^+^CD28^-^ defines effector memory T cells (T_EM_), and CD95^-^CD28^+^ defines naïve T cells (B). The percentage of CD8^+^ T cell expressing T_RM_ markers CD69, CD103, or both CD69 and CD103 were depicted. *, p<0.05; **, p<0.01. One-way ANNOVA compared to PBMC.

PBMC and BAL from AGMs before challenge were used to determine the phenotype of CD8^+^ T cells in peripheral blood and lung respectively at the baseline. Not surprisingly, CD8^+^ T cells in lung exhibited a higher percentage of T_CM_ and T_EM_, but significantly lower levels of naïve T cells compared to those in peripheral blood (Fig 5A). In addition, a significant proportion of CD8^+^ T lymphocytes in lung expressed CD69 (Fig 5B), as previously reported for mucosal T lymphocytes at various mucosal tissues in nonhuman primates [22]. While only a minimal portion of CD8^+^ T cells (mean = 2%) in peripheral blood express the T_RM_ marker CD103, around 20% of CD8^+^ T cells in lung express this marker (Fig 5B). Indeed, around 15% of CD8^+^ T cells in lung express both CD69 and CD103, compared to <1% in CD8^+^ T cells from PBMC (Fig 5B), confirming the existence of T_RM_ in the respiratory compartment of AGMs.

**Fig 6:**
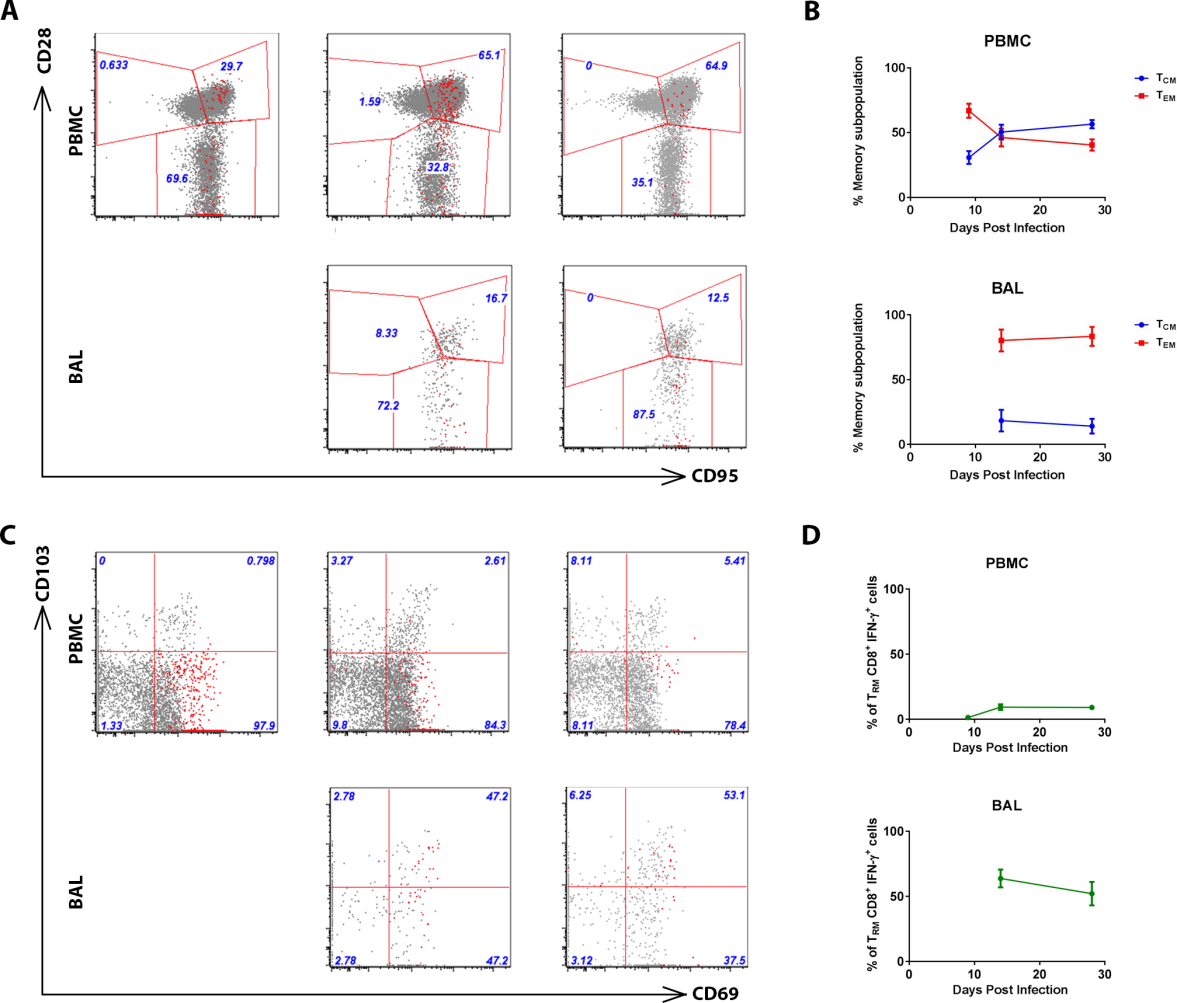
Phenotype of RSV-specific CD8^+^ T lymphocytes in PBMC and in lung. Phenotype of RSV-specific CD8^+^ T lymphocytes in PBMC and in lung in AGMs were analyzed by multi-parameter flow cytometry. (A) Representative data showing the expression of CD95 and CD28 on the surfaces of (N+M)-specific CD8^+^ T cells (IFN-γ-secreting cells, in red) overlaying total CD8^+^ T lymphocytes (in grey) in PBMC and lung of a monkey. Numbers in blue represent percentages of IFN-γ-secreting CD8^+^ T cells falling into the naïve, T_CM_, or T_EM_ gates defined by the expression of CD95 and CD28. The number of (N+M)-specific CD8^+^ T cells in BAL at day 9 were too few to be gated and thus was not analyzed here. (B). Summary of T_CM_ and T_EM_ subpopulations of (N+M)-specific CD8^+^ (IFN-γ-secreting) T cells in all eight monkeys are depicted graphically. (C). Representative data showing the expression of CD69 and CD103 on the surface of (N+M)-specific CD8 T cells (IFN-γ-secreting, in red) overlaying total CD8^+^ T lymphocytes (in grey) in PBMC and lung of a monkey. Numbers in blue represent percentages of IFN-γ-secreting CD8^+^ T cells falling into the quadrants defined by the expression of CD69 and CD103. The number of (N+M)-specific CD8^+^ T cells in BAL at day 9 were too few to be gated and thus was not analyzed here. (D). Summary of CD103^+^CD69^+^ T_RM_ subpopulations of (N+M)-specific (IFN-γ-secreting) CD8^+^ T cells in all eight monkeys are depicted.

We then analyzed the phenotype of RSV-specific CD8^+^ T cells in AGMs in peripheral blood and in lung following infection. While virus-specific CD8^+^ T cells in peripheral blood exhibit a trend of phenotypic evolution from T_EM_ to T_CM_ over time following infection, virus-specific CD8^+^ T cells in lung remained a persistent T_EM_-dominating phenotype without evolution (Fig 6A and 6B). This is consistent to what has been reported previously for T cells at other mucosal sites [22]. Similar to the total CD8^+^ T cells in peripheral blood, only a minimal portion of RSV-specific CD8^+^ T cells in peripheral blood are positive for T_RM_ marker CD103 and CD69. In contrast, around 50% of virus-specific CD8^+^ T cells in lung expressed CD103 and CD69, indicating that these cells reside in lung tissue without circulating (Fig 6C and 6D).

## Discussion

One key challenge for RSV vaccine development is the lack of precise immune correlates of protection since natural immunity generated following RSV infection is not sufficient to protect against subsequent infections in humans. Epidemiological studies have supported different correlates including serum neutralizing antibody titers [13], nasal neutralizing antibody titers [16], nasal IgA [12, 13], mucosal IgG [14], as well as RSV-specific cellular responses [18], depending on what population was studied. In addition, RSV neutralizing antibody titers and F-binding antibody titers were also reported to be inversely correlated with RSV-associated hospitalization [17]. Human experimental infection studies have also demonstrated contradictory results: One adult experimental challenge study demonstrated that infected study subjects had statistically lower serum neutralizing titers and serum IgG titers to F protein [24]. In contrast, another challenge study suggests that mucosal IgA predict better protection from infection [15]. In contrast to humans, naïve AGMs are semi-permissive for RSV infection but seropositive animals are completely resistant to reinfection. Indeed, RSV infection is used as positive control for RSV vaccine studies in AGMs [38–40]. Although AGM is semi-permissive for RSV, sustained viral replication can be observed in lung and nasal epithelium, and sufficient immunological tools are available to allow a meaningful characterization of the immune response against RSV. Therefore in the current set of experiments we evaluated the immunological factors in AGMs that could contribute to protection against RSV infection and compared them to those reported from humans to better inform RSV vaccine studies. RSV infects through the upper respiratory tract mucosal surfaces and normally does not invade systemic circulation, which suggests that efficient local immunity is required and may be sufficient for clearance of infection and possibly prevention of reinfection. Therefore monitoring local responses in the respiratory tract in addition to those in peripheral blood will be critical to understand the protective attributes of the immune response.

We comprehensively characterized primary infection-induced RSV-specific immune responses in naïve AGMs in both peripheral blood and at respiratory mucosal sites where the infection occurs and remains localized. While RSV infection induced humoral and cellular immune responses in both the periphery and respiratory mucosal compartments, mucosal responses were qualitatively and quantitatively different from those in peripheral blood, underscoring the importance of monitoring mucosal responses. Humoral responses in the serum were dominated by IgG and in contrast RSV-specific IgA titers were higher than IgG at mucosal sites (Fig 2C–2E), in agreement with previous reports [41]. The RSV neutralizing antibody titers in AGMs following primary RSV infection were comparable to previous reports [16, 38–40]. The magnitude of the RSV-specific neutralizing antibody responses in AGMs is comparable to those reported in human infants [26] but significantly lower than those in adults (Fig 2B); yet unlike seropositive AGMs, RSV-experienced human adults are susceptible to RSV re-infection [15, 24]. Therefore, although it cannot be excluded that antibody responses could contribute to protection against RSV infection in seropositive AGMs, it is likely that antibodies are not the only contributor towards protection of RSV-experienced AGM. Similar to antibody responses, RSV infection-induced CD4^+^ T cell responses tend to be low in AGMs. In contrast, virus-specific CD8^+^ T cell responses were much more abundant than CD4^+^ T cell responses in peripheral blood and were significantly enriched in the lung. Importantly, the emergence of RSV-specific CD8^+^ T cell responses coincided with the decline of RSV replication / shedding, suggesting a role for virus-specific CD8^+^ T cells in viral clearance. Similarly, in human infants, RSV-specific T cell numbers as well as the total number of activated effector type CD8^+^ T cells peak in blood around day 9–12 after the onset of primary symptoms, i. e., at the time of recovery during primary RSV infection, suggesting the involvement of T cells in the recovery process [42]. Further supporting the role of T cells in RSV clearance, immunocompromised children were shown to suffer more severe diseases and to shed RSV at higher levels for several months, compared with 7–21 days of shedding in healthy children with RSV infection [43].

In addition to quantifying RSV-specific CD8+ T cells in lung and in peripheral blood, we also characterized the phenotype of these cells. The population of RSV-specific CD8^+^ T cells in the lung secreted more cytokines and contained different proportions of T_CM_, T_EM_, and T_RM_ CD8+ T cells compared to counterparts in peripheral blood (Figs 3E and 6). Two populations of virus-specific memory CD8^+^ T cells remain in the circulation after viral clearance. T_CM_ circulate through the secondary lymphoid tissues and encapsulated lymph nodes, using lymph node homing molecules to cross high endothelial venules, and are suggested to be specialized for longevity and proliferation upon reinfection. T_EM_ cells lack lymph node homing molecules and preferentially circulate through the red pulp of the spleen and non-lymphoid tissues, thus surveying body surfaces and visceral organs that are often the initial portal of reinfection [44]. In agreement with previous studies in nonhuman primates showing that vaccine-elicited antigen-specific T cells maintain a persistent and durable T_EM_ phenotype at various mucosal sites [22], we demonstrate that most RSV infection-induced virus-specific CD8^+^ T cells in lung exhibited a T_EM_ phenotype (Fig 6A). In recent years, a new subset of memory T cells termed T_RM_ cells were identified and have triggered interest for their role in local immunity. Shortly after activation in the secondary lymphoid organs, putative T_RM_ seed tissues and undergo differentiation in response to local environmental cues to adopt unique lineage-specific signatures. These long-lived non-circulating T_RM_ cells permanently reside in non-lymphoid tissues including skin, brain, vagina, lung etc. and provide rapid, effective local protection against reinfection relative to circulating counterpart memory T cells [45]. T_RM_ have been identified in mice [37], humans [33] and rhesus monkeys [46]. Consistent with these findings, we demonstrated the existence of T_RM_ CD8^+^ T cells in the lung of AGMs (Fig 5). Moreover, around 50% of RSV-specific CD8^+^ T cells in lung demonstrated a T_RM_ phenotype (Fig 6). Similarly, in an experimental human challenge study, an abundance of CD69^+^CD103^+^ RSV-specific CD8^+^ T cells in lung before infection correlated with reduced symptoms and viral loads, implying that CD8^+^ T_RM_ cells in the human lung play an important role in protection against RSV disease [33]. A recent study also demonstrated that intranasal transferring of airway T cells from RSV-infected mice to naïve mice significantly reduced viral loads in recipient mice upon RSV infection [47]. T_RM_ cells are believed to respond quickly to antigens encountered and act as immune sentinels for reinfection.

Compared to the contribution in viral control / clearance during infection, the potential role of CD8^+^ T lymphocytes in protection against infection has not been well appreciated. However, it has been shown that vaccination through intranasal, but not intraperitoneal immunization of a murine cytomegalovirus (MCMV)-vectored vaccine encoding RSV internal protein M generated robust T_RM_ CD8^+^ T cell responses in lung parenchyma which mediated early antiviral responses and rapidly controlled RSV infection in mice [48]. Similarly, Wu et al demonstrated the contribution of lung T_RM_ cells induced by intranasal vaccination in protection of mice against a lethal intranasal challenge with influenza [49]. In addition, a recent *in vivo* CD8^+^ T cell depletion study revealed the important but unexpected role in live-attenuated trachoma vaccine in mediating protective immunity in rhesus monkeys [50]. Indeed, RSV candidate vaccines affording complete protection in AGMs tend to have T cell components while those eliciting the same or higher level of neutralizing antibodies without detectable T cell responses were not as effective [39]. Most of the current RSV vaccine candidates in the pipeline are deigned to elicit neutralizing antibodies. Our data suggest that an optimal RSV vaccine may also needs to elicit virus-specific CD8^+^ T_RM_ cells.

In summary, our data demonstrate that RSV infection in naïve juvenile AGMs effectively induces virus-specific humoral and cellular immune responses in both peripheral blood and respiratory immune compartments. The magnitude of antibody responses and CD4^+^ T cell responses tend to be low and therefore are unlikely the sole contributor for protection against reinfection. In contrast, RSV-infection induced potent CD8^+^ T cell responses in peripheral blood and enriched CD8^+^ T cell responses in lungs which may contribute to protection against reinfection. RSV-specific CD8^+^T cells in lung exhibit a T_EM_ phenotype and half of them are T_RM_, suggesting that these cells will reside in the local tissue and respond rapidly to a subsequent RSV infection. Additional studies are under-way to explore the role of CD8^+^ T cell responses in protection against RSV infection in seropositive AGMs.

## Supporting information

S1 Fig Multi-parameter ICS flow cytometry data analysis gating strategy and representative plots. (A). Cells were first gated for lymphocytes (FSC-A vs SSC-A) and then analyzed for Live/dead (ViViDye) stain and CD3 expression. Live CD3^+^ T cells were selected for further characterization of CD4 and CD8 expression. CD4^+^ and CD8^+^ T cell subsets were selected and subject to further analysis of cytokine secretion and phenotype / activation markers expression analysis. (B). Representative plots of cytokine secretions including IFN-γ, IL-2, and TNF-α from CD4^+^ and CD8^+^ T cells from PBMC, CD8^+^ T cell from BAL of a monkey with the stimulation of mock or RSV antigens. The percentages of cytokine-secreting cells were gated and quantified.
(TIF)
