# Supplementary figures and images for: Respiratory syncytial virus elicits enriched CD8+ T lymphocyte responses in lung compared with blood in African green monkeys
Source: PLoS One. 2017 Nov 9;12(11):e0187642. doi: 10.1371/journal.pone.0187642 (PMC5679537; doi:10.1371/journal.pone.0187642)

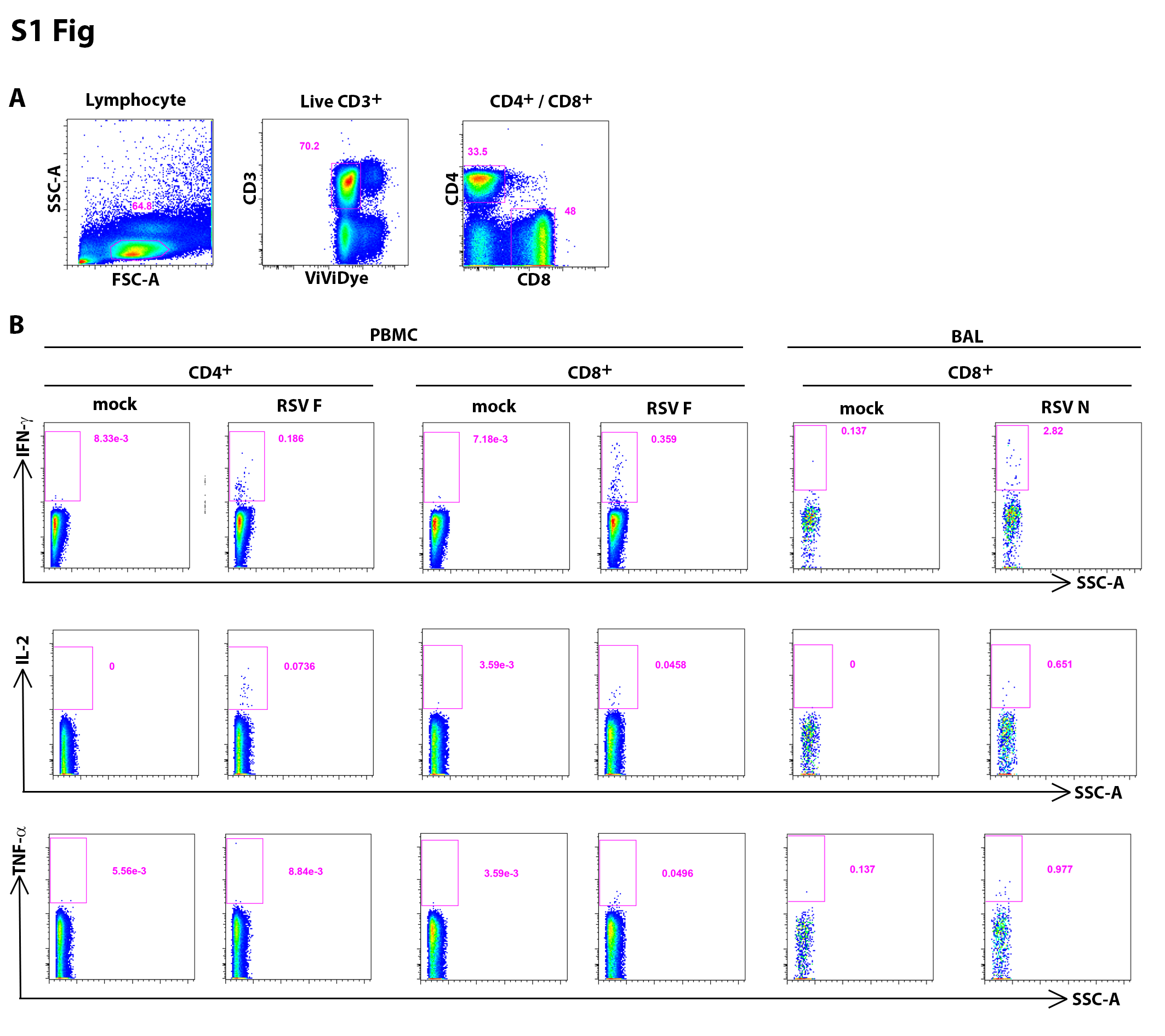

Supplement: S1 Fig — (A). Cells were first gated for lymphocytes (FSC-A vs SSC-A) and then analyzed for Live/dead (ViViDye) stain and CD3 expression. Live CD3+ T cells were selected for further characterization of CD4 and CD8 expression. CD4+ and CD8+ T cell subsets were selected and subject to further analysis of cytokine secretion and phenotype / activation markers expression analysis. (B). Representative plots of cytokine secretions including IFN-γ, IL-2, and TNF-α from CD4+ and CD8+ T cells from PBMC, CD8+ T cell from BAL of a monkey with the stimulation of mock or RSV antigens. The percentages of cytokine-secreting cells were gated and quantified. (TIF) [file pone.0187642.s001.tif]
